# Molecular epidemiological study of *Pseudomonas aeruginosa* strains isolated from hospitals in Brazil by MLST and CRISPR/Cas system analysis

**DOI:** 10.1007/s00438-025-02239-5

**Published:** 2025-03-20

**Authors:** Keyla Vitória Marques Xavier, Ana Carolina de Oliveira Luz, José Wilson Silva-Junior, Beatriz Souza Toscano de Melo, Marcus Vinícius de Aragão Batista, Adrianne Maria de Albuquerque Silva, Valdir de Queiroz Balbino, Tereza Cristina Leal-Balbino

**Affiliations:** 1 Department of Microbiology, Aggeu Magalhães Institute, IAM/Fiocruz, Recife, PE Brazil; 2 Laboratory of Bioinformatics and Evolutionary Biology, Department of Genetics, Federal University of Pernambuco-UFPE, Recife, PE Brazil; 3 Bioinformatics core, Aggeu Magalhães Institute, IAM/Fiocruz, Recife, PE Brazil; 4 Laboratory of Molecular Genetics and Biotechnology, Center for Biological and Health Sciences CCBS, Federal University of Sergipe, Aracaju, SE Brazil

**Keywords:** Mobile genetic elements, MLST, Anti-CRISPR, Phages, Multidrug-resistant

## Abstract

The CRISPR/Cas system defends bacteria and archaea against invasive pathogens, such as phages, establishing an immunological memory from this interaction. *Pseudomonas aeruginosa*, an opportunistic pathogen, represents a significant public health concern due to its multidrug resistance. This study conducted a molecular epidemiological analysis of clinical isolates of *Pseudomonas aeruginosa* in Brazil using multilocus sequence typing (MLST) and characterization of CRISPR/Cas system. Most *P. aeruginosa* isolates harbored the type I-F CRISPR/Cas system (83%), with a subset also exhibiting the type I-E system. Additionally, some isolates presented incomplete CRISPR/Cas systems in their secondary loci. Notably, the isolate Pae93 exhibited a genetic composition rich in phage-related proteins proximal to the orphan CRISPR locus. The identification and characterization of spacer sequences, including previously undocumented ones, revealed a remarkable diversity of predatory mobile genetic elements (MGEs) among the *P. aeruginosa* isolates studied. The spacer sequences were incorporated into the MGE library. Additionally, the study identified the existence of prophages and anti-CRISPR genes. Two new sequence types (STs 3383 and 3384) were identified and added to the PubMLST database. No discernible correlation was established between the observed STs and the previously delineated CRISPR genotypes. However, the CRISPR system remains valuable for elucidating specific interactions between microorganisms and MGEs. The Brazilian population of clinical *P. aeruginosa* isolates was shown to be genetically heterogeneous with a non-clonal distribution, as revealed by MLST analysis. The presence of high-risk clones, such as ST 244 and ST 235, underscores the importance of robust epidemiological surveillance and infection control strategies for *P. aeruginosa*, especially in healthcare settings. This study significantly contributes to the understanding of the molecular epidemiology of these isolates in Brazil.

**Supplementary Information:**

The online version contains supplementary material available at 10.1007/s00438-025-02239-5.

## Introduction

The CRISPR/Cas system is a RNA-mediated adaptive immunity mechanism that protects bacteria and archaea from mobile genetic elements (MGEs). This system includes the Clustered Regularly Interspaced Short Palindromic Repeats (CRISPR) locus, a semi-repetitive genetic structure composed of direct repeats separated by spacer sequences retrieved from MGEs, and the *cas* genes, which encode the system’s enzymatic components (Koonin and Makarova 2019).

This system enables the creation of an immunological and chronological memory of the genetic interactions between the bacterial cell and MGEs, providing an overview of the evolutionary history and environmental conditions of these microorganisms (Luz et al. 2019; Mortensen et al. 2021).

Additionally, the CRISPR locus in some species can also be employed for molecular typing research, even in genetically related isolates, by supporting other molecular typing approaches (Barros et al. 2014; Silva et al. 2023). Given its hypervariability, which is based on prediction and modulation of resistance to phages, the CRISPR locus allows for an analysis of both the content and variability of spacers (Shariat and Dudley 2014; Barrangou and Dudley 2016).

*Pseudomonas aeruginosa* is an opportunistic pathogen, globally recognized as one of the main factors causes of hospital-acquired infections, particularly affecting immunocompromised patients (Kunz Coyne et al. 2022). According to the WHO (2024), the growing number of multidrug-resistant (MDR) pathogens and the emergence of resistance to last-resort treatment options make this bacterial species a significant public health issue.

In Brazil, antimicrobial resistance is not yet fully understood. However, studies have shown that hospital-acquired infections are the main transmission route for these pathogens, with significant consequences for the country (Silva et al. 2012; Padoveze and Fortaleza 2014; Braga et al. 2018). Notably, studies report the prevalence of *Pseudomonas aeruginosa* in healthcare-associated infections (HAIs) in different Brazilian states (Lisboa et al. 2007; Braga et al. 2018; Rodrigues et al. 2020; Figueredo et al. 2021), along with the presence of high-risk clones such as ST111, ST244, and ST235, among others. Additionally, ST277, an endemic clone highly associated with isolates producing metallo-β-lactamases (SPM-1), has been reported in Brazil (Dos Santos et al. 2023; Rodrigues et al. 2024).

Carbapenem resistance in Brazilian *Pseudomonas aeruginosa* isolates has been widely reported. Rodrigues et al. (2020) identified that 66% of the analyzed isolates carried β-lactamase-encoding genes and exhibited a high prevalence of multidrug-resistant (MDR) and extensively drug-resistant (XDR) phenotypes. The dissemination of carbapenemase-producing strains significantly increased during and after the COVID-19 pandemic, likely due to hospital overcrowding, inadequate medical team preparedness, and the indiscriminate use of antibiotics during this period. Additionally, MDR phenotypes have also been detected in strains isolated from hospital sewage, including *P. aeruginosa* (Zagui et al. 2020), reinforcing the importance of this pathogen as a public health concern. These findings highlight the need for a deeper understanding of the spread of these strains and the population dynamics of *P. aeruginosa* in Brazil, contributing to more effective strategies for antimicrobial resistance monitoring and control.

Given the increasing incidence of infections caused by multidrug-resistant (MDR) *P. aeruginosa*, alternative therapeutic approaches are of utmost importance. One alternative strategy is phage therapy, which involves the administration of specific phages to target pathogenic bacteria (Monteiro et al. 2019). Despite its promising results, understanding how bacteria respond to phage invasion and how the CRISPR/Cas system functions in pathogens are crucial for the development of effective therapies (Kakasis and Panitsa 2019; Mortensen et al. 2021).

The CRISPR/Cas system, by blocking the entry of mobile genetic elements (MGEs), holds great potential as an ally in controlling the spread of resistance genes through horizontal transfer. Additionally, the CRISPR locus, being a hypervariable region associated with the prediction and modulation of phage resistance, allows for detailed analyses of spacer content and variability, even among genetically related isolates. This characteristic makes CRISPR a strategic target for molecular epidemiology studies (Shariat and Dudley 2014; Barrangou and Dudley 2016).

## Materials and methods

### Bacterial strains and storage conditions

The study analyzed a total of 174 *Pseudomonas aeruginosa* isolates. Among them, 12 clinical isolates were selected from an initial set of 120 samples, collected from different infection sources in patients and sectors of two public hospitals in Recife, Pernambuco, Brazil. These isolates were previously screened for the presence of the CRISPR/Cas system using qualitative PCR (Luz et al. 2019), and only CRISPR/Cas-positive isolates were chosen for the investigation.

Additionally, 13 CRISPR/Cas-positive isolates previously collected from the same hospitals were included, totaling 25 clinical isolates analyzed in this study. To further expand the analysis of the genetic diversity and molecular epidemiology of the species, 149 Brazilian isolates available in the PUBMLST database were incorporated, resulting in a total of 174 isolates in the investigation (Table 1).

**Table 1:** Isolation data on *Pseudomonas aeruginosa* clinical isolates used in the present study

Isolate	Year	Units of the hospitals	Clinical sample	Hospital
Pae90	2016	Ward	Urine	H1
Pae93	2016	Ward	Urine	H1
Pae94	2016	ICU	Tracheal aspirate	H1
Pae104	2010	General surgery	Blood	H2
Pae105	2010	Cardiology	Urine	H2
Pae107	2010	ICU	Blood	H2
Pae108	2010	Cardiology	Tracheal aspirate	H2
Pae110	2010	General clinic	Wound secretion	H2
Pae114	2010	General clinic	Wound secretion	H2
Pae115	2010	Surgery clinic	Urine	H2
Pae116	2010	Cardiology	Wound secretion	H2
Pae127	2010	Vascular clinic	Catheter bridge	H2

The isolates are stored in the Department of Microbiology, Aggeu Magalhães Institute, IAM/Fiocruz/PE, preserved in BHI (Brain Heart Infusion) and 30% glycerol (1:1) at −80 °C.

### Genome sequencing

Genomic DNA of the 12 isolates was extracted with DNeasy Blood and Tissue Kit (Qiagen) following the manufacturer’s instructions. Libraries were prepared with Nextera XT Library Preparation Kit (Illumina) and quantified through real-time PCR with NEBNext Library Quant Kit for Illumina (New England Biolabs) and the QuantStudio (Thermo Fisher Scientific). The libraries were sequenced on the MiSeq platform (Illumina) using the MiSeq Reagent Kit V3 with 600 cycles (Illumina).

### Genome assembly and annotation

The quality of the reads obtained was assessed with FastQC software. Genome assembly was conducted using Unicycler (Bankevich et al. 2012). Annotation was performed using the Prokka software (Seemann 2014). Genome assemblies were visualized with Artemis software (Rutherford et al. 2000), and proteins of interest were compared using Basic Local Aligment Search Tool (BLAST tool) against the Uniprot database to confirm their identity.

### CRISPR/Cas system and spacers analysis

Confirmation of CRISPR/Cas systems in the genomes were conducted with CRISPRCasFinder (Couvin et al. 2018). Origins of spacers found in CRISPR loci were determined with CRISPRTarget (Biswas et al. 2013). The identified spacers were compared to those available in a spacer library published previously (Luz et al. 2019).

### Phage and anti-CRISPR genes investigation

Bacteriophage search in the genomes of *P. aeruginosa* isolates of type I-F was conducted using PHASTER (Zhou et al. 2011). The phage areas identified by PHASTER were verified using BLAST (Johnson et al. 2008), by comparing these regions with the genomes of the two most likely phages that PHASTER identified.

PHASTER assigns scores to the phage regions, considering different criteria such as overall phage length and related structures (tail and capsid). Prophages were only considered present if 1) PHASTER results indicated phage region integrity as intact or 2) PHASTER results suggested questionable integrity, but BLAST results returned an alignment coverage percentage of 50% or more. The bacteriophage sequences identified by PHASTER were compared with the spacers obtained, to determine whether the isolates possess self-targeting spacers.

For a more comprehensive characterization of the content observed in the orphan locus in Pae93, the search for homologies between this region and prophages was also conducted using Prophage Hunter (Song et al. 2019), VIBRANT (Kieft et al. 2020), and DBSCAN-SWA (Gan et al. 2022). The presence of anti-CRISPR genes in the prophages was investigated with BLAST (NCBI database) by comparing with sequences of 14 previously described anti-CRISPR genes (Pawluk et al. 2014, 2016, 2018).

### Determination of isolate genetic profiles and phylogenetic analyses

Sequence types (STs) were determined with Multilocus Sequence Typing (MLST) technique using the online tools of the specific *P. aeruginosa* database on PubMLST (https://pubmlst.org/paeruginosa/) (Jolley et al. 2018).

A phylogenetic tree was generated using a dataset composed of concatenated sequences of the seven MLST genes from 12 isolates collected in the present study, along with 13 previously collected isolates (Luz et al. 2019), totaling 25 isolates. Additionally, 149 Brazilian *Pseudomonas aeruginosa* isolates available in PUBMLST were included (Supplementary Material 3). An evolutionary model was selected for the concatenated genes using the jModelTest 2.1.10 software.

Based on the selected parameters, a Maximum Likelihood (ML) phylogenetic tree was then constructed using the RAxML-NG software (Kozlov et al. 2019). Branch support was performed with 1,000 bootstrap replicates. ML tree search was optimized, and 10 random starting trees were used. The phylogenetic tree was viewed and edited using iTol v.6 (Letunic and Bork 2019).

### Molecular epidemiology by CRISPR/Cas

Among the 25 CRISPR/Cas positive isolates from hospitals 1 and 2, only those with the I-F system were chosen to map the CRISPR-based dissemination pathway. Due to the prevalence of type I-F over other types, the focus was solely on this type to expand the sample size, ensure a homogeneous sample, and avoid false comparisons. Thus, the spacers of *P. aeruginosa* isolates were classified and organized into subtypes as previously described previously (Luz et al. 2019) Genotypes were established based on the spacer content, where as little as one different spacer would be indicative of a new genotype (Shariat and Dudley 2014).

### Statistical analyses

To assess the agreement between typing methods (ST, CRISPR genotype, and CRISPR Groups), the adjusted Wallace statistic was employed (Severiano et al. 2011). The analysis aimed to determine the degree of predictive concordance among the different methods. Adjusted Wallace values were computed for all method combinations, and 95% confidence intervals (95% CI) were determined using the bootstrap method to ensure result accuracy.

### Ethical approval

Not applicable. The present work did not involve humans, human specimens, or tissues. Bacteria were obtained purified from the hospitals per request.

## Results

The confirmation of CRISPR/Cas system types revealed a higher frequency of the type I-F system (83%) followed by the type I-E system (17%) in the 12 initial genomes examined (Table 2). Most isolates that containing both type I-F and I-E systems often showed two CRISPR loci in their genomes. Examining isolates Pae90, Pae93, Pae107, and Pae108, however, revealed that, in contrast to the first locus, the second CRISPR locus “lacked a complete CRISPR/Cas system, missing the *cas* genes (Table 2).

**Table 2:** CRISPR/Cas types identified in the studied *Pseudomonas aeruginosa* Brazilian isolates

CRISPR/Cas types	Isolate
I-F	Pae105; Pae112; Pae114; Pae115; Pae116
I-E	Pae94
I-F and LES	Pae90; Pae108; Pae110
I-E and orphan	Pae93

**Fig. 1:**
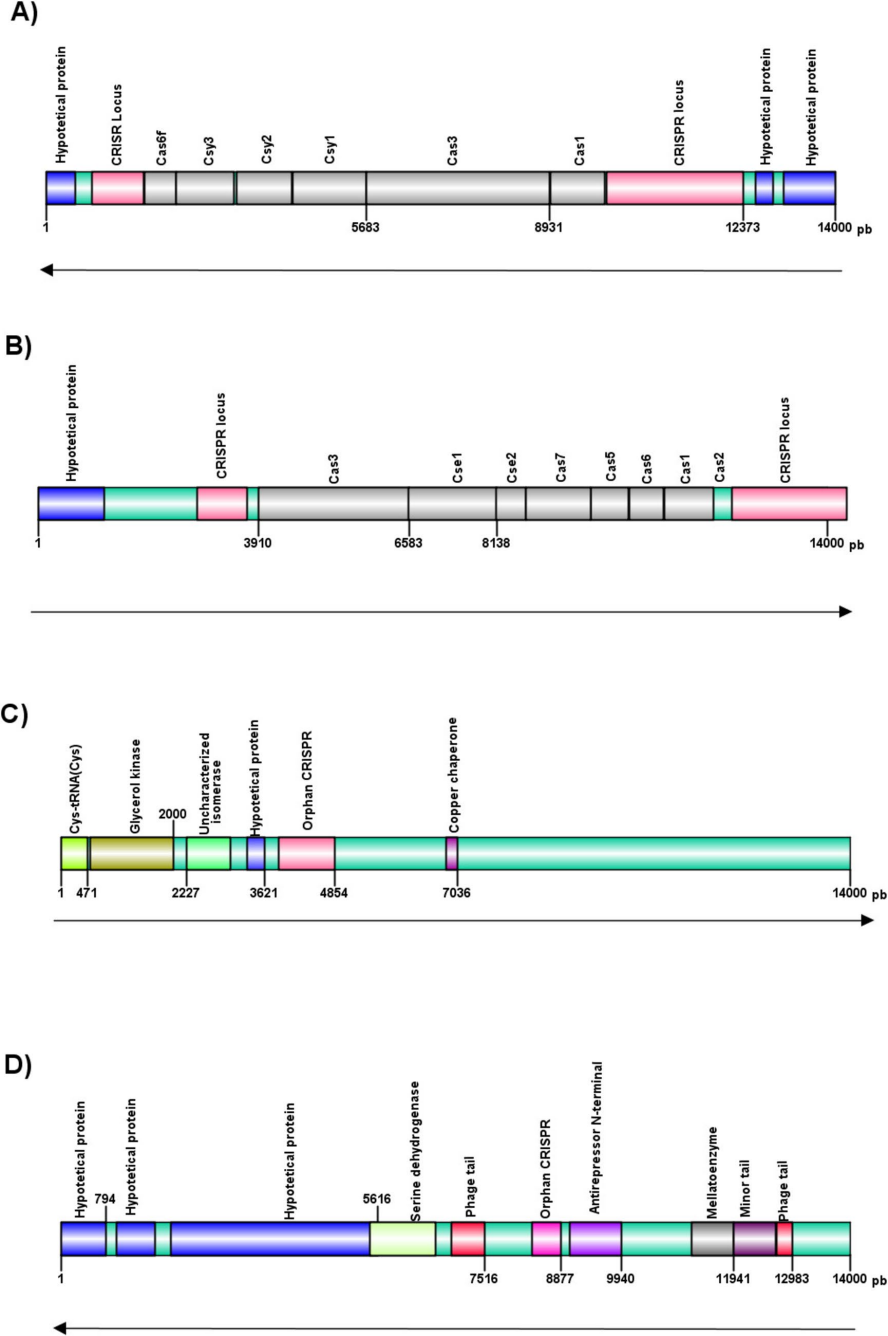
Variations of the CRISPR/Cas System in Brazilian clinical isolates of* Pseudomonas aeruginosa*: **A** Type I-F; **B** Type I-E; **C** Orphan CRISPR/Cas system; **D** Genetic context observed in isolate Pae93

The isolates presenting a CRISPR locus lacking *cas* genes shared the same gene arrangement, with the exception of Pae93 isolate (I-E) (Fig. 1). This isolate exhibits a genetic composition rich in phage-related proteins adjacent to the orphan CRISPR locus.

Based on the results from VIBRANT, a CRISPR locus encoded by a phage was identified inserted into the genome of Pae93. BLAST analysis of this genomic area against the NCBI viral database (Brister et al. 2015), the AUS531 Phi phage or the YMC11 phage are the most likely phages in the region, with the highest identity (94%). DBSCAN results also indicate the presence of phage-associated proteins encoded in regions adjacent to the analyzed locus, with higher identity (98%) to the YMC11 phage. In contrast, Prophage Hunter did not identify this viral region, but proteins associated with the YMC11 phage were detected.

### Analysis of spacers

The current spacer library, updated from the earlier version (Luz et al. 2019), now has 1231 spacers, 267 of which are spacer sequences that were acquired from the isolates that were the subject of the current investigation. Of these, 120 are considered novel, as they had not been previously described, and 149 had already been documented (Supplementary Material 1).

**Fig. 2:**
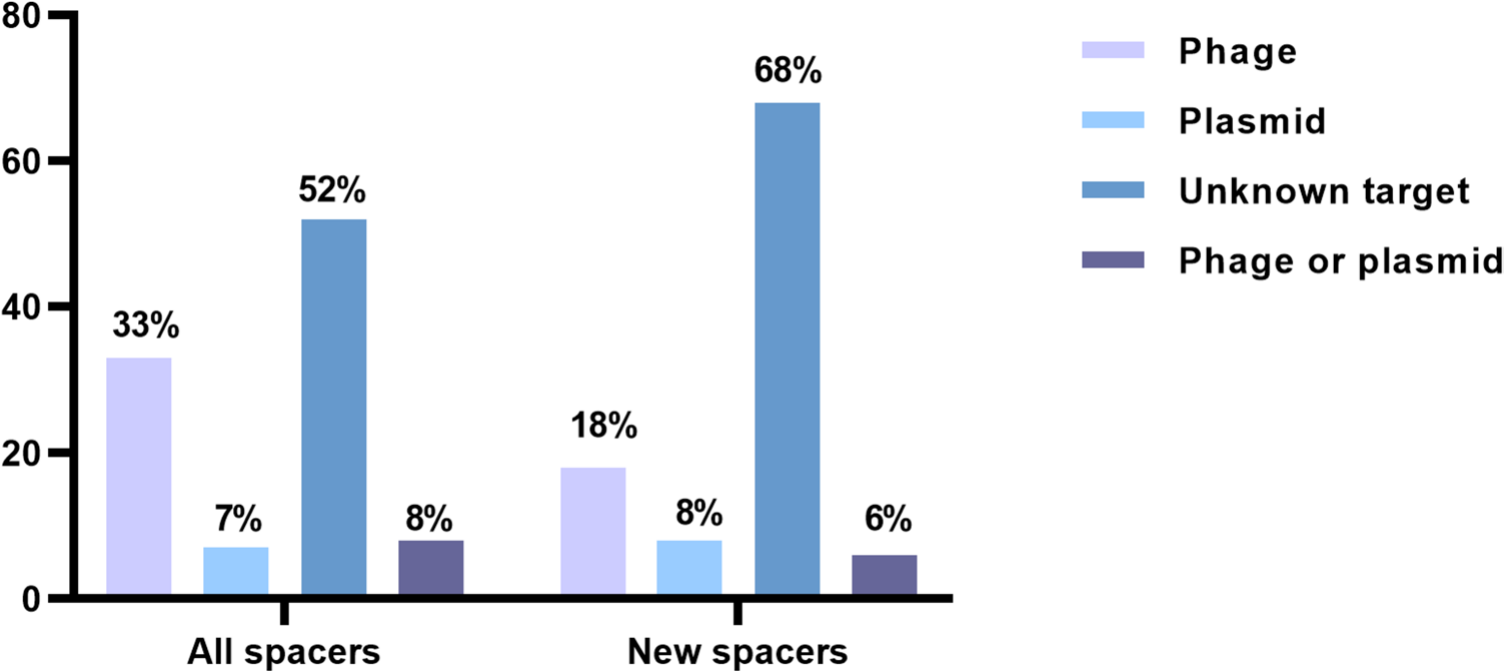
Percentage of spacer sequences and their potential targets. In blue, the percentage of potential targets of the spacer sequences in general is observed. In orange, the relationship between the 120 never-before-seen spacer sequences and their potential targets is observed

Of the 267 spacers, 52% had no similarity with any known MGEs and were classified as ‘unknown’. Among the remaining sequences, 40% of spacers exhibited homology with phages or plasmids, while 8% showed sequence similarity to both phages and plasmids simultaneously (Fig. 2) (Supplementary Material 2).

### Prophages and anti-CRISPR genes

The presence of spacer sequences directed targeting prophages was analyzed for the 12 genomes studied (Table 3). All isolates were found to have one or more prophages in their genomes, but no corresponding spacer sequences were identified.

**Table 3:** Prophages in the genomes of *Pseudomonas aeruginosa* under study

Prophage	Isolates									
	Pa90	Pa104	Pa105	Pa107	Pa108	Pa110	Pa114	Pa115	Pa116	Pa127
Pf1	*			*	*	*				*
JBD44	*									
YMC11	X	X	X						X	X
JBD25		X	X				*	X	X	
F10		*	*						*	*
PAJU2				*						
PHAGE_Escher_vB_EcoM				*			*	*		
PHAGE_Shigel_SfII					*					
phi2					X					
phiCTX						*			*	
phi297						*			X	
PHAGE_Shigel_Sf6						*	*	*	*	
MD8								*		
phi3										*

Prophages marked with “x” have target spacers, while prophages marked with “*” do not

To better understand the incorporation of these prophages into the *P. aeruginosa* samples, a search for anti-CRISPR (*acr*) genes was conducted. Despite the presence of prophages in all genomes, only three isolates carry *acr* genes: Pae114 and Pae115, which encoded acrf6, and Pae116, which encoded acrf7.

### Molecular epidemiology

The analysis of the profile of the 12 isolates identified nine different STs, seven of which had been previously deposited in the PubMLST database, while two new STs (STs 3383 and 3384) were submitted to PubMLST. Following the definition of STs, a molecular epidemiological analysis based on MLST and CRISPR was conducted to understand the spatiotemporal distribution of *P. aeruginosa* I-F clinical isolates from a region in northeastern Brazil, originating from hospitals 1 and 2 (Table 4).

**Table 4:** Epidemiological characteristics of the 25 Brazilian clinical isolates of *Pseudomonas aeruginosa*

Isolate	ST	Month	Year	Hospital	Units of the hospitals	Clinical sample
Pae 12	244	Jun	2015	H1	Ward	Bone fragment and soft tissues
Pae 74	244	May	2016	H1	ICU	Sputum
Pae 104	244	April	2010	H2	General surgery	Blood
Pae 105	244	April	2010	H2	Cardiology	Urine
Pae 127	244	Oct	2010	H2	Vascular clinic	Catheter tip
Pae 22	252	June	2015	H1	ICU	Sputum
Pae 70	275	May	2016	H1	Ward	Urine
Pae 29	357	July	2015	H1	ICU	Sputum
Pae 42	357	Mar	2016	H1	ICU	Sputum
Pae 116	360	July	2010	H2	Cardiology	Wound secretion
Pae 94	620	June	2016	H1	ICU	UTI
Pae 114	850	July	2010	H2	Cl	Wound secretion
Pae 115	850	July	2010	H2	General clinic	Urine
Pae 108	1076	May	2010	H2	Cardiology	Tracheal aspirate
Pae 90	1239	June	2016	H1	Ward	Urine
Pae 21	1394	June	2015	H1	Ward	Urine
Pae 110	2234	July	2010	H2	General clinic	Wound secretion
Pae 66	3078	April	2016	H1	Ward	Bone fragment
Pae 28	3079	July	2015	H1	Ward	Surgical material
Pae 39	3079	July	2015	H1	ICU	Sputum
Pae 83	3079	June	2016	H1	ICU	Bone fragment
Pae 81	3080	May	2016	H1	Ward	Urine
Pae 113	3137	July	2010	H2	Ward	Bone fragment
Pae 93	3383	June	2016	H1	Ward	Urine
Pae 107	3384	May	2010	H2	ICU	Blood

Isolates with the genetic profile ST 244 were observed in hospitals 1 (H1) and 2 (H2) during the years 2010, 2015, and 2016 (Table 5). In H1, ST 244 was first discovered in the ward environment in June 2015 and later isolated from the ICU in May 2016 from samples of soft tissues, bones, and secretions. In H2, isolates with ST 244 were obtained from various hospital departments, including the cardiology ward, vascular clinic, and surgery, as well as from blood, urine, and catheter tip samples. These isolates exhibited a pattern of intra-hospital dissemination, initially observed in H2 in April 2010 and subsequently in H1 in 2015 and 2016. The presence of ST 244 in both hospitals and across different clinical sectors suggests inter-hospital spread of this clone.

**Table 5:** Genetic characterization based on the CRISPR/Cas system of local isolates of *Pseudomonas aeruginosa*

Isolate	ST	CRISPR genotype	Group
Pae12	244	G9	E
Pae74	244	G11	E
Pae105	244	G13	E
Pae127	244	G10	E
Pae29	357	G3	B
Pae42	357	G16	B/E
Pae116	360	G14	E
Pae104	620	G12	E
Pae114	850	G4	C
Pae115	850	G5	C
Pae110	2234	G15	E
Pae66	3078	G1	A
Pae28	3079	G6	D
Pae39	3079	G7	D
Pae83	3079	G8	D
Pae113	3137	G2	A
Pae107	3384	G17	E/D

As of the year 2010, isolates from H2 showed potential clonal dispersals of ST 244 and ST 850. ST 850 was isolated in the clinical and surgery sectors, originating from wound secretions and urine, respectively. ST 3079 was observed in H1 in July 2015, in both the ward and ICU sectors, isolated from surgical material and sputum, respectively. In June 2016, the same clone continued to be present in patients’ sputum in the ICU, further suggesting intra-hospital dispersion.

To explore the potential the potential of the CRISPR locus in *P. aeruginosa* as a genotyping tool compared to the MLST technique, an initial sample consisting exclusively of isolates harboring the type I-F system was selected for analysis. This sample consisted exclusively of isolates harboring the type I-F system, collected from hospitals H1 and H2 (totaling 17 isolates). The analyses focused on the arrangement of spacers and the formation of groups based on differences in spacer content among the isolates. These groups were then compared with the STs obtained by MLST to assess the consistency between the two techniques. It was observed that isolates belonging to Group E are associated with four different STs (244, 360, 620, 2234), while Group A comprises two STs (3078, 3137). The remaining groups showed genotypic uniformity, each represented by a single ST.

The analysis of spacers yielded 17 distinct genotypes, each corresponding to a *P. aeruginosa* isolate. However, the molecular typing based on spacer content did not reveal a strong correlation with the STs established by MLST.

Based on the adjusted Wallace statistic and the 95% confidence interval (95% CI), the obtained value of 0.826 indicates a moderate concordance between ST and Group typing, suggesting that knowing the ST provides a reasonable probability of predicting the Group. The confidence interval (0.652–1.000) shows that this relationship is statistically significant and can vary between moderate and strong. However, the concordance for defining the ST from the Group was low (0.240), indicating that knowledge of the Group has limited predictive ability regarding the ST. The correlation between ST and CRISPR was 0.000, suggesting no predictive capability of ST regarding the CRISPR genotype and vice versa. The confidence interval (0.000–0.000) confirms this lack of correlation, indicating a null relationship between the two typing methods. This discrepancy underscores the need for further investigation into the utility and effectiveness of CRISPR as a genotyping tool compared to conventional MLST techniques.

**Fig. 3:**
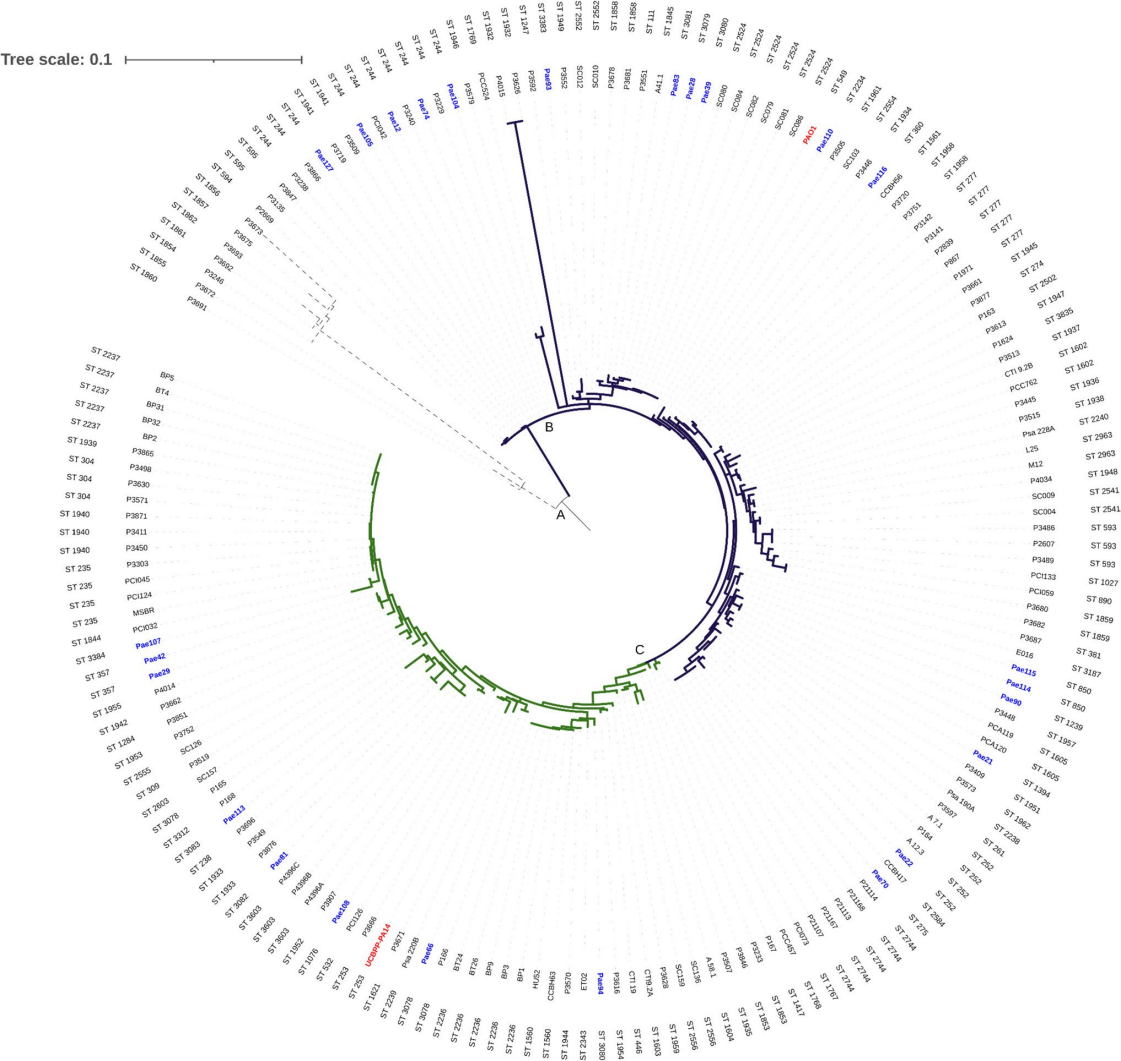
Phylogenetic tree of 174 Brazilian isolates of *P. aeruginosa.* In the tree, group A is delimited in dashed black. Group B is delimited in navy blue, and group C in green. Isolates are identified in the innermost circle of the tree, with the 25 isolates collected from hospitals H1 and H2 highlighted in blue. In red, the PA01 and PA-14 strains used as reference strains are highlighted. The corresponding STs of each isolate are provided and positioned in the outermost circle of the tree

A phylogenetic analysis was conducted for the isolates collected in hospitals H1 and H2, based on the concatenated sequences of MLST alleles, as well as sequences from Brazilian isolates deposited in the PubMLST database (Fig. 3) (Supplementary Material 3).

The Maximum Likelihood phylogeny (ML) of the 174 sequences yielded a huge number of unique branches, which has to do with the absence of required information to define the branching order of the examined isolates. A total of 109 different STs were observed in the *P. aeruginosa* population studied, indicating a non-clustered distribution of Brazilian clinical isolates.

Three major groups were formed based on ML. Group A has a smaller number of isolates, characterized as the outgroup. Group B comprises a larger number of isolates, including most of our isolates and the reference strain PAO1. Group C is formed by 69 isolates, with the reference strain for CRISPR studies, UCBPPA-14, as its representative.

When the sequences from hospitals 1 and 2 were compared to sequences from the PUBMLST database, it was discovered that, despite the majority of samples having a non-clonal distribution, these isolates were grouped with those from other hospitals. Additionally, there is a holistic distribution of our isolates on the tree. Few isolates were grouped into clonal complexes.

Particularly, ST 244 formed a clonal complex (CC) with ST 1941, sharing six common MLST alleles. P3229, collected in 2008 from hospital effluent, is more closely related to Pae74 and Pae104, collected in 2016 and 2010 from different human samples, than to Pae12, an isolate collected in the same hospital as Pae74 but in different years. Pae105, collected in the cardiology sector, is the sister group of PCI042, isolated in 2007 from a blood sample. Although Pae105 and Pae127 have the same characteristics, such as the same hospital, collection year, and ST, there are a genetic distance between them.

Isolates collected between 2000 and 2002 form CC 277. Blood samples from P2839, P869, and P1971 were collected, and as they form a cluster with similar characteristics, it can be assumed that this clone has spread over time.

Pae114 and Pae115 isolates were collected in 2010 from H2, originating from different clinical samples, and together with E016, isolated in 2013, they form a clonal complex. These isolates share three common MLST alleles with isolate P3687, collected in the year 2010 from hospital effluent. Thus, it is believed that these variations in alleles are influenced by the environment, allowing the bacteria to better adapt to their surroundings.

The uniform distribution of samples allowed for the examination of evolutionary and recombination dynamics among Brazilian isolates. Pae113, collected from tissue fragments in 2010, shares three MLST alleles with P168, an isolate collected from water in 2018. The allele sharing and the formation of sister groups are also observed in Pae81 and P3907, where despite sharing only two MLST alleles, both were collected from urine in 2016 and 2011, respectively.

Isolates with the genetic profile ST 3603, collected in 2017 from blood samples, as well as isolate Pae81 (ST 3008), collected in 2016 from a urine sample, although having different STs, form a clonal complex (CC) as they share six common MLST alleles. Additionally, isolates Pae66 and P166, which both belong to the same clonal complex (ST 3078), share alleles with Psa 220B, P367, and UCBPPA-14.

## Discussion

The frequency of system types I-F and I-E, and the genetic structure of the two CRISPR/Cas systems are already well established in *P. aeruginosa*, as well as the presence of orphan CRISPR (Van Belkum et al. 2015; England et al. 2018; Luz et al. 2019). Particularly, the Liverpool epidemic strain (LES), globally recognized for its presence in North America and Europe infecting cystic fibrosis patients, presents an intact type I-F CRISPR arrangement, well conserved, but lacking associated *cas* genes. This suggests a partial ancestral loss of the system, rendering it non-functional. The absent or non-functional CRISPR might still allow strains to acquire and maintain virulence through mobile genetic elements (Van Belkum et al. 2015).

Orphan CRISPR was observed in clinical isolates of *P. aeruginosa* of the I-F type in our study. The presence of orphan CRISPR was also observed in Brazilian clinical isolates harboring the I-F system but it was not possible to establish if this genetic context is inherently linked to the I-F system (De Oliveira Luz et al. 2021). The presence of spacer sequences in these orphan loci may provide adaptive immune memory to their hosts, enhancing their protection against infections by competitor phages, if they are found concurrently with complete CRISPR/Cas systems (Al-Shayeb et al. 2020).

The spacer library is a strategic tool for understanding the MGEs that interact with *P. aeruginosa*, where these sequences can reveal connections between circulating strains, contributing to a better understanding of epidemiological dynamics and enhancing strategies for tracking and controlling nosocomial infections. Additionally, the spacer sequences can influence *P. aeruginosa*’s ability to acquire additional resistance genes or participate in transformation events, both of which have a significant impact on antimicrobial resistance. Furthermore, these sequences may assist in the development of alternative therapies, such as phage therapy, by providing data for the selection of potential lytic phages, as well as in CRISPR/Cas-based therapies, either by preventing the acquisition of resistance genes or by sensitizing bacteria to antibiotics (Bikard et al. 2012; Gholizadeh et al. 2020).

Phages carrying the CRISPR/Cas system have been observed previously (Seed et al. 2013; Al-Shayeb et al. 2020, 2022), and the absence of *cas* genes from the CRISPR/Cas system in phages is quite common. The phage lifecycle is generally coordinated from a host response. Repressor/antirepressor viral proteins are responsible for maintaining the lysogenic cycle or inducing entry into the lytic cycle of these temperate phages (Engelhardt et al. 2015). Specifically, the presence of the antirepressor protein (Ant) from the prophage carrying a CRISPR locus in Pae93 emphasizes another mechanism of interaction and dynamics between phage and host (Silpe et al. 2020).

The anti-CRISPR proteins AcrF6 and AcrF7 are classified within the *aca1* gene class, which deactivate the CRISPR/Cas system during the interference phase (Pawluk et al. 2016). This finding is consistent with the outcomes of our investigation, where in the Pae116 strain exhibited specific spacers targeting bacteriophages JB25, YMC11, and Phi297, while the Pae115 strain possessed specific spacers for phage JBD25. Hence, the presence of such specific spacers against the mentioned phages should theoretically prevent their entry, were it not for the presence of genes that inhibit the system.

The presence of anti-CRISPR genes in the *P. aeruginosa* genome indicates competition between phages and bacteria (Pawluk et al. 2018). Despite identifying phages in genomes, some self-targeting spacers against them, as well as the presence of *acr* genes responsible for inactivating the CRISPR system, the locus structure remains intact in genomes.

Regarding to other prophages found, it has been observed that phages that do not have the ability to inactivate the CRISPR/Cas system through anti-CRISPR genes can take advantage of phages that possess this capability, through cross-reactivity, as the spacers show homologies to a certain number of commonly related but also genetically distant targets (Chevallereau et al. 2020).

It has been observed that, although there are phages capable of inactivating the CRISPR/Cas system, such inactivation does not occur immediately. Thus, a concentration of abortive phage attempts is required until the quantity of *acr* genes reaches a sufficient level to deactivate the defense mechanism (Borges et al. 2018).

In a previous study conducted with clinical Brazilian isolates of *P. aeruginosa*, the presence of anti-CRISPR genes in the genomes was also observed, inferring that the system in these isolates would be inefficient due to the presence of phages inserted in the genome, as well as the presence of a spacer with homology to a gene specific to the bacterium, responsible for encoding oxygen-independent coproporphyrinogen III oxidase (Luz et al. 2019).

Although there are differences in the results between CRISPR and MLST, it is essential to recognize the unique evolutionary path of each microorganism. Addressing the CRISPR/Cas system from this perspective provides a deeper understanding of the relationships between these microorganisms and Mobile Genetic Elements (MGEs) on an individualized scale. This suggests that, despite potential incongruences in the genetic profiles observed through CRISPR compared to MLST, studying the CRISPR system remains crucial for unraveling the complex interactions between microorganisms and MGEs. Furthermore, future studies may consider a larger number of samples, with a more comprehensive inclusion of isolates from different geographical regions and hospital environments, to deepen the understanding between MLST and CRISPR, as discussed by Barros et al. (2014) for *Yersinia pestis*, Van Belkum et al. (2015) for *P. aeruginosa*, and Silva et al. (2023) for *Acinetobacter baumannii*.

The phylogenetic analysis allowed the evaluation of the population distribution of 174 Brazilian *Pseudomonas aeruginosa* isolates based on MLST. Of the 109 STs identified among the isolates, 76 are not shared, meaning they correspond to unique STs present in specific isolates. This distribution reinforces the results, showing a non-clonal population structure and significant genetic diversity in the sequences. The high frequency of recombination between the alleles examined is evidenced by the presence of few clones in this pathogen, a characteristic commonly observed in *P. aeruginosa* (Oliver et al. 2015; Nageeb et al. 2021).

Withing the increase in multidrug-resistant (MDR) and extensively drug-resistant (XDR) strains of *P. aeruginosa*, also known as high-risk clones, in hospital settings has raised significant concerns for global public health (Chen et al. 2014; Oliver et al. 2015; Dou et al. 2017; Fang et al 2022). Among the sequences analyzed, the presence of high-risk clones was confirmed among Brazilian clinical isolates, such as ST 244 (n = 10) and ST 235 (N = 4).

ST235 is globally linked to an MDR/XDR phenotype, associated with over 60 variants of β-lactamase, such as classes A and B. This virulence is also associated with genetic determinants of homologous recombination (DprA), increasing the ability to acquire and spread resistance genes when compared to other clonal complexes, besides not possessing the CRISPR/Cas system (Treepong et al. 2018; Del Barrio-Tofiño et al. 2020; Fischer et al. 2020).

The presence of this clonal complex in patients with urinary tract infections has been reported in the literature (Park and Koo 2022; Cottalorda et al. 2022). One of the strains in the phylogeny (PCI124) was collected from a urinary tract infection in 2008, indicating the need for further studies to understand if there is a possible correlation also in Brazilian isolates. Therefore, the monitoring and development of new strategies to prevent the spread of this clone are globally important.

ST277 is an endemic clone of Brazilian Pseudomonas aeruginosa strains, strongly associated with an MDR/XDR profile and the production of the SPM-1 metallo-β-lactamase (blaSPM-1), the primary mechanism of carbapenem resistance identified in Brazilian clinical multidrug-resistant isolates. Studies have demonstrated a direct association between this ST and the presence of the Type I-C CRISPR/Cas system, carried by an Integrative and Conjugative Element (ICE) (Belkum et al. 2015; Silveira et al., 2020). Reports on the occurrence of this clone in other countries are scarce; however, countries such as the United States, Australia, and China have identified and deposited it in PubMLST, signaling a warning about the dissemination of an ST linked to numerous resistance genes (De Oliveira Santos et al. 2019).

ST244 has been identified as one of the most frequent clonal complexes among Brazilian clinical isolates of *Pseudomonas aeruginosa*, with its high prevalence also reported in cities in China (Chen et al. 2014; Zhao et al. 2023). Similar to ST235, ST244 is strongly associated with resistance to carbapenems, the last-line treatment for infections caused by MDR Gram-negative bacteria (Fang et al. 2022; Sakurai et al. 2023).

The clinical relevance of high-risk clones is linked to the fact that these STs are associated with severe infections in immunocompromised patients, causing pneumonia, bacteremia, urinary tract infections, and even sepsis (Moradali et al. 2017). Carbapenem resistance is mediated by the production of β-lactamases, porin alterations, efflux pumps, and the acquisition of resistance determinants through mobile genetic elements (Kos et al. 2015; Li et al. 2015; Fang et al. 2022).

The global spread of these clones poses a significant challenge to public health, due to its direct impact on the effectiveness of available treatments and the increase in infections in healthcare settings. Previous studies (Kiffer et al. 2005; Empel et al. 2007; Chen et al. 2014; Fang et al. 2022; Sakurai et al. 2023) highlight the presence of resistance in these and other clones over the years in various locations around the world. These data underscore the importance of epidemiological and genetic studies for the monitoring and control of these isolates, as well as the need for the responsible use of antimicrobials and the development of alternative therapies to curb this spread and the problems caused by bacterial resistance.

This study underscores the importance of the CRISPR/Cas system in the evolutionary dynamics of *P. aeruginosa*, highlighting its interaction with mobile genetic elements. Despite the disparities between CRISPR and MLST, investigation into this system remains pivotal for comprehending the intricate relationships between microorganisms and MGEs. Furthermore, the molecular epidemiological analysis shed light on the structural and population diversity of Brazilian isolates of *P. aeruginosa*, emphasizing the imperative for the monitoring and control of these isolates to safeguard global public health.

## Conclusion

This study provides valuable insights into the molecular epidemiology of clinical *Pseudomonas aeruginosa* isolates in Brazil. Our findings emphasize the need for the implementation of genomic surveillance programs in hospital settings to monitor, control, and adopt preventive measures to curb the spread of high-risk clones due to their clinical impact on antimicrobial resistance. Notably, the CRISPR system stands out as a promising tool in epidemiological investigations, capable of tracking genomic evolution and microbial interactions, enabling a deeper understanding of the population dynamics in closely related strains. Furthermore, the study revealed a genetically heterogeneous population of clinical *P. aeruginosa* isolates in Brazil, with the presence of clinically important clones such as ST244, ST235, and ST277, reinforcing the need for continuous efforts to mitigate the challenges associated with antimicrobial resistance.

## Supplementary Information

Below is the link to the electronic supplementary material.

Supplementary file1 (XLSX 61 KB)

Supplementary file2 (XLSX 83 KB)

Supplementary file3 (XLSX 76 KB)

## Data Availability

The genomes sequences of the 12 *Pseudomonas aeruginosa* isolates Pae90, Pae93, Pae94, Pae104, Pae105, Pae107, Pae108, Pae110, Pae114, Pae115, Pae116 and Pae127 sequenced in this study have been deposited in the National Center for Biotechnology Information (NCBI) database under the BioSample accession: SAMN31839910; SAMN31839911; SAMN31839912; SAMN31839913; SAMN31839914; SAMN31839915; SAMN31839916; SAMN31839917; SAMN31839918; SAMN31839919; SAMN31839920; SAMN31839921, respectively. The BioProject ID is PRJNA514718.
